# A Review of the Use of Gaze and Pupil Metrics to Assess Mental Workload in Gamified and Simulated Sensorimotor Tasks

**DOI:** 10.3390/s24061759

**Published:** 2024-03-08

**Authors:** Holly Gorin, Jigna Patel, Qinyin Qiu, Alma Merians, Sergei Adamovich, Gerard Fluet

**Affiliations:** 1 Rehabilitation and Movement Sciences Department, Rutgers School of Health Professions, Newark, NJ 07107, USA; hg284@shp.rutgers.edu (H.G.); patel421@shp.rutgers.edu (J.P.); qiuqi@shp.rutgers.edu (Q.Q.); merians@shp.rutgers.edu (A.M.); sergei.adamovich@njit.edu (S.A.); 2 Biomedical Engineering Department, New Jersey Institute of Technology, Newark, NJ 07102, USA

**Keywords:** cognitive workload, cognitive effort, oculometrics, pupillometry

## Abstract

Gaze and pupil metrics are used to represent higher cognitive processes in a variety of contexts. One growing area of research is the real-time assessment of workload and corresponding effort in gamified or simulated cognitive and motor tasks, which will be reviewed in this paper. While some measurements are consistent across studies, others vary and are likely dependent on the nature of the effort required by the task and the resulting changes in arousal. Pupil diameter is shown to consistently increase with task effort and arousal; however, the valence of arousal must be considered. In many cases, measures of pupil diameter were sensitive to both excessive and insufficient challenge. Overall, it is evident that gaze and pupil metrics are valuable to assess the cognitive state during gamified and simulated tasks, and further research is indicated regarding their use in clinical populations in rehabilitation to inform optimally engaging interventions.

## 1. Introduction

The importance of an adequate quantity, duration, and intensity of rehabilitation to maximize motor and functional recovery post neurologic insult is well established [1]. Yet, research has indicated that mere participation in a high dose intervention is insufficient to drive neuroplasticity and recovery. Kleim and Jones [1] outlined the principles of experience-dependent neuroplasticity and indicated that patients not only need high repetitions of intense therapeutic activity, but also need to be motivated and engaged during the intervention process. Rehabilitation programs that are both challenging and engaging are essential to facilitate the optimal task dosage and salience. One increasingly popular mode of therapy that increases engagement and adherence is the use of virtual reality (VR)-based gaming simulations [2]. However, even in this training environment, the level of patient engagement and motivation is not always discernible or measurable. While there are many self-reporting and subjective methods to assess the engagement of a patient and their intrinsic motivation to perform a certain task [3,4], like any subjective measure, they are subject to increased biases and intrusive to administer mid task. In addition, patients who are post neurologic insult do not always have the verbal or written communication abilities required to complete the subjective measures. Thus, there is a need for more objective and quantifiable measures of patient engagement and effort.

The important question is: can we determine whether a patient is cognitively engrossed, engaged, and motivated by an activity? Researchers use physiological measures, including cardiac and electrodermal autonomic metrics, to assess the cognitive state and effort during gaming experiences [5]. With improvements in technology, eye and pupil metrics have become more readily accessible and reliable, and, as a result, research demonstrates the correlation with cognitive and affective states as well [6]. While eye and pupil measures have been used to quantify the difficulty and degree of effort in a variety of tasks, there is limited research on their use in a rehabilitation setting in clinical populations. Virtual reality-based training provides an optimal medium for use of these metrics to assess motivation and engagement, due to the ease of tracking gaze and pupil metrics. In addition, immersive virtual reality-based training provides the highly controlled and customizable aspects of an experimental environment with the freedom of movement and ‘natural’ environment of the real world [7]. Even non-immersive virtual reality simulations allow for simpler and more specific changes in task parameters compared with real-world tasks, which is important for assessing the patient experience in different interventions to determine optimal task parameters and promote engagement.

Thus, the use of gaze and pupil metrics to assess the cognitive state during virtual reality interventions is potentially invaluable to the design of therapeutic tasks that optimize engagement, neuroplasticity, and recovery, and warrants further investigation. This paper will review the neurophysiology of gaze fixation, saccades, blinking, and pupil dilation as it relates to cognitive workload and engagement in virtual and simulated activities.

## 2. Materials and Methods

An initial set of searches was performed in PubMed. Keywords searches that combined each of the individual terms: “pupil dilation”, “saccades”, “fixation”, “blink”, “gaze metrics”, “pupillometry”, or “oculometrics”, with each of the individual terms: “engagement”, “effort”, and “workload”, using the Boolean operator AND to produce 21 unique searches. These 21 searches produced 4734 articles. One reader independently assessed these studies for the following inclusion criteria: (1) published in English; (2) conducted between 2000 and 2023; (3) studied gaze or pupil dilation metrics in any population; (4) evaluated higher cognitive processes or cognitive workload; (5) involved any task with a gamified component, motor activity (with or without a simulated functional task), or the use of virtual reality. This resulted in six primary studies and two review papers that were included for review.

A set of follow-up searches were performed using the six included primary articles: (1) all the authors of each included study were searched for on Google Scholar for additional studies; (2) all the reference lists were searched for by hand for additional qualifying studies; and (3) articles citing qualifying studies were searched for using Google Scholar in an attempt to find additional qualifying studies. This search yielded nine primary studies and two additional reviews that were included for review. See Table 1 for all the included primary studies.

## 3. Results and Discussion

### 3.1. Saccades

Gaze-orienting eye movements include quicker, ballistic movements, known as saccades or slower smooth pursuit movements. While saccades can abruptly change the direction of the eye movement, smooth pursuits are primarily voluntary and serve to maintain a moving image on the fovea. Saccades can be voluntary or reflexive and vary from large amplitude gaze shifts to significantly smaller microsaccades. Cranial nerve control of extraocular muscles directs all eye movement, voluntary or reflexive [23]. It is important to note that, while these measures are not discussed in this review, vergence and vestibulo-ocular movements serve to stabilize the eye. In addition, even during visual fixations, the eye is in continual motion, characterized as smooth ocular tremors and ocular drift, as well as very low amplitude saccades, known as microsaccades that fall under the same neuronal control as traditional saccades [24]. Additional reflexive eye movements, often mistaken for microsaccades, include ocular following responses, which are very short latency (~70 ms in humans) reflexive eye movements triggered by the sudden, unpredictable motion of large textured patterns in the visual field. They are thought to represent the initial components of vestibulo-ocular and, possibly, smooth pursuit movements. While they are extremely small in amplitude and accordingly difficult to measure, ocular following responses have been successfully tracked in both children and adults [25].

Saccades in areas of interest are controlled by gaze centers in the reticular formation, the paramedian pontine reticular formation, which controls horizontal gaze, and the rostral interstitial nucleus in the mesencephalic reticular formation, which controls vertical gaze. These gaze centers activate or inhibit cranial nerve motor nuclei, which serve to innervate muscles both ipsilaterally and contralaterally, resulting in eye movement to the encoded location. The amplitude of movement is dependent on the duration of activity of these oculomotor neurons. The two gaze centers receive input from the superior colliculus and the frontal eye fields, both of which contain a retinotopic map; the upper motor neurons here are activated by sensory stimuli, and their firing results in extraocular muscle activity and eye movement needed to align the fovea with the coded retinotopic coordinates. The superior colliculus is thought to mediate reflexive saccades, while the frontal eye fields can activate the superior colliculus or the gaze centers directly for more voluntary saccades [23]. While the neuronal control of smooth pursuits is not as well understood, similar neuronal structures and circuitry are at play [26].

Common saccadic movement metrics include the number of saccades, the saccade average velocity, the saccade peak velocity, the saccade amplitude, the saccade duration, and the saccade frequency. In response to increased mental workload and task demand, some studies found a decrease in the saccade measures of amplitude, duration, peak velocity, and frequency. Marandi et al. [22] investigated the reliability of gaze metrics over days using a standard computer-based task. Subjects viewed an arrangement of points that created a shape, memorized their sequence, and then recreated the shape following a washout period. The saccade measures decreased with increased task difficulty. Likewise, Tao’s 2019 systematic review of driving and pilot simulation studies reported a decrease in saccade measures with increased cognitive workload [27]. Di Stasi’s work with a gamified firefighting task and driving simulator also resulted in decreased saccade peak velocity with increased difficulty [9,10]. In contrast, Mallick’s tetris study found an increase in peak saccade velocity with increased game speed [15], and Skaramagkas’s general review reported a trend of increasing saccade velocity with increases in cognitive workload and visual attention demands [6]. Other studies, such as a VR language comprehension task by Schirm, found no difference in saccade measures across difficulty levels [19].

Di Stasi discussed the discrepancy in saccade velocity response to increased workload in the literature and proposed that it is due to the varying impact of increased task difficulty on arousal [9]. Hu et al. also explored this idea when reviewing the use of eye metrics to identify active (mental) fatigue versus passive (sleepiness) fatigue in drivers [28]. While it is reasonably well established that saccade velocity decreases with passive fatigue and decreased arousal induced from a prolonged time on task, there are inconsistent results in response to increased task difficulty. It was proposed that this discrepancy is due to the characteristics of the task and its demands, such as motivation and associated rewards. Di Stasi and Hu speculated that, while some increases in task difficulty may result in increased mental arousal with no mental fatigue and increased saccade velocity, other increases in task demands may result in mental fatigue with decreased arousal and resulting decreased saccade velocity. For example, in Di Stasi’s firefighting task, saccade peak velocity was measured after participants had spent two hours on a task and suggested that the decreased velocity reflected mental fatigue. Of note, the majority of these studies evaluated saccade speed via peak velocity over an average velocity, and one study that evaluated both found a significant difference in peak velocity between workloads but not in average velocity [10]. Consistent with this, Di Stasi discussed that, because peak velocity measures are not affected by saccade detection thresholds, it is a stronger measurement than average velocity [9].

While the causes of decreased saccade velocity with fatigue and decreased arousal are not well understood, one theory centers around central fatigue: the loss of muscle force with fatigue attributed to decreased central motor cortical output [29]. This is well established in skeletal muscle following both physical exercise [21] and, while not as well supported, mentally demanding tasks [30,31]. Evidence supports that this is mediated by widespread changes in neurotransmission, involving serotonin, dopamine, and norepinephrine [29]. Connell et al. demonstrated decreased saccade velocity following fatiguing physical exercise, showing the impact of central fatigue on the oculomotor system. This decreased velocity was reversed with caffeine and norepinephrine–dopamine reuptake inhibition, implicating the dopaminergic and noradrenergic systems [32,33]. To our knowledge, there is no study demonstrating the impact of central fatigue induced by mental (rather than physical) exertion on saccade measures. Perhaps mentally fatiguing tasks inducing central fatigue, such as in Aguilera’s study [29], result in decreased motor output and resulting decreased saccade velocity as well. This is an area requiring further research.

An increase in saccade velocity with increased task difficulty associated with higher arousal, motivation, and reward seeking, as proposed by Di Stasi, may be mediated by dopaminergic mechanisms related to reward. The link between dopamine, striatal neurons, and reward-motivated activities is well established [34]. There is evidence linking dopamine with the control of saccades [35,36,37], and researchers use saccades as indicators of basal ganglia function [38]. Behaviors can be extrinsically motivated, driven by a tangible reward or outcome, or intrinsically motivated, driven by internal satisfaction generated by the task itself [39]. Success in gamified or simulated tasks can be intrinsically motivating for subjects, providing that there is a reward that inspires an increased effort to improve game performance as the task demands increase. This may underlie the increase in saccade velocity sometimes seen with increased task demands.

Saccade velocity, specifically peak velocity, was the most commonly used measure to classify mental effort and fatigue. The nature of the task, in this case its impact on arousal and fatigue, impacts the resulting changes in saccade velocity. This evidence supporting the use of saccade velocity to evaluate the cognitive state in existing gamified and simulated tasks supports its use in rehabilitation to assess the engagement and arousal of patients in response to different therapeutic tasks.

### 3.2. Fixation

Ocular fixation is the maintenance of gaze on a single location and serves to focus images on the fovea of the retina, the point of the densest photoreceptors and highest visual acuity. Researchers measure many characteristics of fixation, including the number of fixations, the fixation frequency (in total or in an area of interest (AOI)), the fixation duration, the total fixation duration on an AOI, fixation density, the time to first fixation, and repeat fixations [6]. This review focuses on fixation frequency and fixation duration, as the reviewed studies used these fixation measures most frequently.

Neuronal control of visual fixation is still under active investigation, but research indicates that it is controlled by the same structures that control eye movements [24]. Omnipause neurons in the paramedian pontine reticular formation, noted above for its role in horizontal saccades, increase their firing during fixation and are hypothesized to mediate the required inhibition of saccades to allow for fixation to occur. These omnipause neurons also receive input from the superior colliculus. While superior colliculus activity generally results in eye movement as described above, some neurons in the part of the SC representing the foveal region of the retina conversely display increased activity during fixation. The balance between these neurons across the superior colliculi maintains fixation, and a disruption in this balance triggers saccades to fixate on the location represented by the active neurons on the superior colliculus map. The bilateral balance of activity is then restored to maintain fixation on the new area of interest [24], which also requires a tonic level of corresponding cranial nerve firing to hold the static position of the eye [23]. The medio-posterior cerebellum is also involved in fixation control, as well as regions in higher order frontal and parietal cortex.

Cognitive and perceptual load can affect fixation duration and frequency in response to increased mental workload in different ways. Perceptual load, the amount of stimulus-driven (bottom-up) processing of sensory information, can vary separately from cognitive load, which is the deliberate top-down modulation of this information and the use of higher cognitive processes [40]. Changing the number of items or the number and complexity of the perceptual processes required to complete a task manipulates perceptual load [41]. In contrast, manipulations such as adding a secondary memory or mental imagery task increases the cognitive load. When the perceptual requirement exceeds the perceptual capacity, the viewer is not able to process all sensory stimuli, resulting in shorter, more frequent fixations as they attempt to process the entire scene. When the cognitive load of a task exceeds capacity, the ability to filter relevant versus irrelevant stimuli decreases, resulting in longer and fewer fixations as each fixation processes surplus sensory information [14].

Aware of the inconsistent findings of gaze responses to increased task demand in the literature, Liu et al. aimed to distinguish the impact of increased cognitive versus perceptual load using a puzzle-solving video game. As expected, increases in cognitive load resulted in increased fixation duration and decreased fixation frequency, while increases in perceptual load caused the duration to decrease and the frequency to increase [14]. Many other studies of gamified and simulation tasks have followed this trend. Subjects in Marandi’s study demonstrated an increase in fixation duration and a decrease in fixation frequency as the number and complexity of points to memorize increased. Schirm’s VR language comprehension study found a decrease in fixation frequency and an insignificant trend towards increased duration at their optimal difficulty level [19]. Marandi’s shape memory task and Schirm’s language comprehension task resulted in gaze fixation changes that were consistent with increases in cognitive demand. In contrast and consistent with increased perceptual load, Mallick et al. found a decrease in duration and an increase in frequency in response to increased speed in a tetris game [15]. Di Stasi et al. asked subjects to perform a simulated firefighting task based on two different strategies. While one was primarily stimulus-response driven and required the subject to identify and compare the size of different fires (perceptual load), the other required strategic prediction and a top-down approach (cognitive load). The fixation frequency was not evaluated, but the prediction strategy (cognitive load) game elicited a higher gaze duration than the more perceptually demanding version [9].

While many studies are consistent with this cognitive versus perceptual load divide, a systematic review of cognitive demand during surgical simulations and procedures reported an increase in both fixation duration and frequency with increased workload [42]. A 2019 systematic review by Tao et al. of physiological measures of mental workload across multiple domains, a large percentage of which were driving and pilot simulation studies, found that the fixation duration decreased with task demand, but the fixation frequency only increased in 50% of the reviewed studies [27]. Skaramagkas’s 2023 general review reported a consistent increase in fixation frequency (5/5 studies) to increased cognitive workload, and varying results in fixation duration (two showed increases, three showed no change, and six showed decreases). The same review separated visual attention from cognitive workload and, in studies evaluating visual attention, found an increase in both the fixation duration and the number of fixations with increased attentional demands [6].

Across studies, results on fixation duration and fixation frequency vary, and this discrepancy is in part due to the lack of classification of the cognitive versus perceptual load of each task. Nevertheless, these studies provide evidence that quantifying gaze fixation and duration can be a useful tool when attempting to measure engagement and motivation, thus supporting its potential value as a tool to assess patient response to virtual rehabilitation and inform task parameters that maximize engagement and recovery. However, to our knowledge, there are no studies that use these measures in a neurologic rehabilitation setting. Therefore, studies are needed to confirm whether these measures function in a similar fashion in patients with neurological dysfunction.

### 3.3. Blink

Blinking is both a voluntary (intentional blinking) and involuntary (spontaneous and reflexive blinking) process. Blinking serves many purposes, including hydration of the cornea and protection of the eye from external damage. Spontaneous blinks are the most common, and occur unconsciously, approximately 15 times a second, and generally in the absence of inciting stimuli [43]. Humans blink spontaneously more frequently than required to maintain eye lubrication, and spontaneous blinks are linked to cognitive processes and attention [44]. Similar mechanisms and muscles mediate the mechanical output of all types of blinks and are controlled by brainstem structures [43]. While reflexive blinks are maintained as a brainstem circuit between the fifth and seventh cranial nerves in response to perturbation of the eye, the timing of spontaneous blinks involves a somatosensory network in the upper cortical structures. Visual cortical areas are also intricately involved in mediating uninterrupted vision, despite the occlusion of the eye to visual stimuli during a blink [43]. The frequency of eye blinks is the most common blink measurement in the context of cognitive state [6]. Of note, eye trackers use light illumination to track gaze (see ‘Equipment and processing’), and this can result in the drying of the cornea and increased blinking frequency. However, this effect is primarily observed with the use of visible white light over non-visible infrared light [45], and the majority of modern eye trackers use infrared light. Nevertheless, it is important to consider this potential impact when interpreting blink frequency data.

Most studies and reviews have found that blink frequency consistently decreases with increased task demand and arousal, and increases with drowsiness [15,27,28,42,46], although some studies found no differences [19,22]. However, like gaze fixation, there is speculation about the impact of task demand on blinking metrics, specifically, tasks that require internally directed attention cause increased blinking and tasks that require externally directed or visual attention cause decreased blinking. In Schirm’s VR language task, it was hypothesized that there would be an increase in both blink frequency and duration, due to evidence that the internal cognition required of the task would result in decreased engagement with the external environment and increased blinking to ‘block out’ irrelevant environmental input; however, they ultimately found no difference between the difficulty levels [19]. The internal nature of the language comprehension task in this VR study contrasts with many of the other gamified and simulation-based tasks reviewed here, underlying this conflicting hypothesis. Mallick also discussed the influence of external visual processing demands versus internal cognitive workload on blink duration and correctly hypothesized that the dual visual and cognitive demands of tetris would result in a decrease in blink duration regardless [15]. Skaramagkas’s review found a trend towards decreases in blink frequency (4/5 studies) with increased cognitive workload and visual attention demands [6].

There is additional evidence that spontaneous blink rate increases with tasks requiring internally directed attention, such as maintaining information in working memory. One study found different changes in blink frequency during different components of a visual memory task. Blink rate was lowest when subjects were committing a visual image to memory and highest during the delay period when subjects were maintaining information in their working memory. In contrast, another study showed no difference in eye blink frequency in tasks with an internal vs an environmental focus [47]. Supporting an increase in blink rate with internally directed attention, blink frequency increases with the activity of the dorsal attentional network and decreases with the activity of the default mode network: two distinct neural networks that mediate tasks requiring external vs internal attention, respectively [48].

Another hypothesis suggests that working memory causes increased dopamine [44,48], which is responsible for the resulting increased blink rate [44]. Cognitively demanding tasks, such as working memory functions, demonstrate an inverted U-shape relationship with dopamine, where either too much or too little impairs performance [44]. In line with this, conditions with decreased dopamine, such as Parkinson’s disease, result in decreased blink rate, while increased dopamine conditions result in increased blink rate. Eye blink rate is commonly used as a proxy for striatal dopamine levels [49], although this correlation is not found in all studies [50]. Consistent with the established link between dopamine and reward anticipation, eye blink rate often also predicts performance in reward-incentivized tasks [49,51]. Similarly to the measure of saccade velocity, perhaps an increase in intrinsic reward seeking is responsible for the increased blink rate with increased task difficulty. 

It is evident that blink frequency can provide insight into gamified and simulated task demand and may even discern between internally and externally directed effort. This supports its value as a potential clinical indicator during rehabilitation and points to the need for future research.

### 3.4. Pupil

The autonomic nervous system mediates changes in pupil size; sympathetic activation causes pupil dilation, and parasympathetic activation causes pupil constriction via the sphincter and dilator muscles located in the iris. The pupil constricts in response to increased luminance and near fixation. Parasympathetic pupil constriction is mediated by the Edinger–Westphal nucleus, which directs motor output to the iris sphincter muscle via the third cranial nerve. Pupil dilation occurs with increased arousal or mental effort [52]. It is mediated by the hypothalamus and locus coeruleus, which project to the intermedio-lateral column of the spinal cord, triggering motor output to the iris from the superior cervical ganglion. The locus coeruleus also inhibits the pupil constriction pathway [52]. It is well established in research settings that pupil dilation increases with task effort and arousal in healthy individuals [6,8,11,13,15,16,18,19,20,21,22,27,28], as well as in individuals with CNS pathology, including stroke [8] and Parkinson’s disease [12]. In cases where researchers evaluate multiple eye metrics, pupil diameter is often reported as a more consistent marker of cognitive effort compared with other measures [6,15,16].

Because pupil dilation likely reflects a ‘basic physiological aspect of processing load that is independent of qualitative differences between tasks’ [53], pupil responses can represent increased effort and arousal across a wide variety of tasks [52,53]. That being said, one limitation of using pupil diameter to assess mental workload may be its lack of specificity. While it is responsive to the intensity of mental workload, it offers no information about the valence (positive or negative nature) of the arousal or the type of task demand [14,52,54]. This is what motivated Liu et al. [14] to investigate fixation measures as a complementary measure to pupil dilation to improve the identification of task demand. In addition to cognitive workload, pupil dilation also increases with emotional arousal [6,11,18] and other higher cognitive processes, making it difficult to discern which specific process is responsible for the pupil response.

Task-evoked pupillary responses are an averaged pupil size over the performance of a task, occurring 100–200 ms after the onset of processing and subsiding quickly once the processing ends [53]. Pupillary responses from other factors, such as emotional stimuli, have longer lasting effects and impact the baseline pupil diameter; collecting a baseline measurement helps to control for these factors [17,53]. Pupil diameter is also impacted by many other factors, including lighting and luminance changes [55], mental health conditions [56], age [55], refractive errors [55], sleep deprivation [57,58], and certain prescription and over-the-counter drugs, including caffeine and alcohol use and withdrawal [59,60,61,62]. Researchers often instruct and inquire about recent sleep quantity and drug use of their subjects prior to data collection.

Researchers look at many pupillary measures, including mean pupil diameter, peak pupil diameter, latency to the peak, and percentage change in pupil size. Because of the impact of illumination levels on pupil diameter, it can be difficult to discern the cause of the change in pupil size, especially when luminance is not recorded and reported. Many studies do note that illumination was held constant in all trials, or discuss the possible confounds of varying illumination levels. The Index of Cognitive Activity (ICA) is a pupil metric that acts to differentiate between pupil size changes caused by light vs cognitive processing. While pupil responses to light are more gradual, the ICA counts the number of more rapid instances of pupil dilation, which are attributed to cognitive activity. Rather than comparing averaged pupil diameters between conditions and with a baseline, which is the case in the majority of studies discussed here, the ICA measures the moment-to-moment rate of change of in pupil diameter and is growing in popularity [63].

As an added note highlighting the significant relationship between neurological function and normal pupil behavior, the Neurological Pupil Index (NPi) is used as a prognostic indicator in acute care settings. Post neurological insult, the pupillary light reflex is consistently assessed and used as a prognostic indicator of neurological function and recovery, as abnormal pupil function accompanies worsening cerebral edema, intracranial hypertension, and impending brain herniation. Generally, this is clinically assessed via subjective assessment, leaving it vulnerable to inaccuracies and decreased inter-rater reliability. The Neurological Pupil Index (NPI) is a quantitative pupil measure acquired with automated infrared pupillometry to mitigate these limitations. The NPI considers several variables, including pupil size, percentage, latency, velocity of constriction, and velocity of dilation, and uses an algorithm to produce a score from 0–5; values under 3 represent an abnormal NPI [64]. The NPI has been shown to be a valuable prognostic tool to predict unfavorable long-term outcomes post acquired brain injury [64,65].

Although it is true that pupil dilation is not specific to one cognitive domain or type of task demand, there is evidence that it is responsive to both insufficient challenge, causing decreased arousal and boredom, and to excessive challenge, potentially causing cognitive overload and frustration. Many studies have shown that pupil responses increase linearly with task demand [8,15,16,17,20,22]. Other studies demonstrate maximal pupil dilation occurring not at the highest level of difficulty, but perhaps at the optimal level of difficulty when compared with the abilities of the user [13,19,20,21], as described by the Challenge Point framework [66]. For example, in a pilot simulation task where the number of flight indicators was changed to manipulate difficulty, the medium (and not high) difficulty level elicited significantly higher pupil dilation compared with the low difficulty and control levels, while the pupil dilation during the high difficulty level fell (non-significantly) below the medium level [21]. Similarly, Strauch et al. manipulated the difficulty of the game pong in two different ways by changing either speed or paddle size. For manipulations of paddle size, pupil dilation increased linearly with difficulty level, and for changes in speed the maximal pupil dilation occurred during the medium difficulty level compared with low and high difficulty [20]. These results are consistent with research aimed at investigating the flow experience, a state of comprehensive immersion and engagement in a task that occurs at the peak of an inverted U shape, involving autonomic processes and arousal versus difficulty level rather than at the highest point in a linear response [67,68]. Lu et al. reported a positive linear relationship between the flow state and pupil dilation using a gamified version of a memory task [14]. This sensitivity of pupil dilation to both underchallenging and overchallenging tasks makes it potentially very useful in real-time task modification to achieve optimal difficulty; in fact, many investigators have attempted to do just this with machine learning algorithms during gaming [20,69].

The function of this arousal pupil response is unknown and under investigation. One theory frames it using situations of arousal of negative valence, such as danger, and suggests that the change in pupil size functions to balance visual sensitivity (increased by pupil dilation) and visual acuity (decreased by pupil dilation). Some suggest that it is simply a side effect of neural processes and serves no function [52]. Interestingly, much like blink rate, increased pupil dilation is tied to dopamine and reward-based tasks [70,71,72]. Interestingly, in a study that compared pupillary response with increased postural demand in healthy individuals and individuals with Parkinson’s disease (a condition with decreased dopamine), the subjects with Parkinson’s disease demonstrated greater increases in pupil dilation. However, Parkinson’s disease also causes motor symptoms that result in decreased postural control, requiring increased effort to withstand increased postural demand than healthy individuals. While postural demand appears to differ from other gamified or computer-based tasks as primarily physically taxing, there is a high degree of cognitive effort and executive functioning skills required to initiate the correct strategies at the right time [12].

Overall, pupil dilation is the most consistent marker of increased cognitive arousal and effort with increases in task difficulty. Again, this measure should be further investigated in clinical populations during rehabilitation interventions.

### 3.5. Equipment and Processing

The majority of modern eye trackers are video based and most commonly utilize an infrared or near-infrared light. The eye tracker then identifies both this reflection of the light off the cornea and the center of the pupil. Through a calibration process, where participants look at a series of predetermined points of known screen location, the gaze tracker can then use the relative positions of the pupil and corneal reflection to pinpoint the gaze location. The different trackers vary by sampling rate, with frequencies (measured in Hertz (Hz)) ranging from collecting approximately 50 up to 2000 gaze points per second. Many different systems exist, including both table-mounted (positioned in front of the participant) and head-mounted systems. Some table-mounted systems require the use of a chin rest to stabilize the position of the head when high levels of precision are required (i.e. reading small text). A 2020 review by Carter et al. cites Tobii and SR Research as the leading manufacturers, both of which use infrared technology and offer both table-mounted and head-mounted devices [73]. Some more advanced systems combine eye tracking with the tracking of other facial features, introducing the recognition of emotional facial expressions and responses while performing different tasks, widening the use in psychological research. Announced in 2023, the Apple Vision Pro is a modern VR system equipped with 12 cameras and five sensors that offers high resolution tracking of gaze and emotional responses [74]. When considering the equipment used in the reviewed studies, there is a lot of variance in the brand of eye tracker, with more than 10 trackers mentioned throughout the reviewed studies and no consensus on a gold standard (see Table 2). The majority were stationary eye trackers positioned in front of the participant, with the exception of Schirm’s VR phone booth study [19], which used a headset, Kahya’s postural demand study [12], which used glasses, Di Stasi’s studies, which used head-mounted equipment [9,10], and some pilot and driving simulation studies discussed in secondary articles. The sampling frequency ranged from 60–500 Hz.

While some studies include details of the pre and post processing of eye data to retrieve the desired metrics and/or definitions, and the criteria of each metric with referenced recommendations, such as in [8,18,19,22], the details vary widely across studies. As oculometrics and pupillometry grow in popularity, many algorithms continue to be developed and enhanced that process eye tracker data and extract the desired metrics [7,75,76], though there is no gold standard. Some, but not all, of the reviewed studies specified that the heads of the participants were free to move or, more rarely, the use of a chin rest [20]. Overall, there is no consensus or widely adopted standardized methodology or practice, and authors across the reviewed studies using diverse equipment and methods achieved significant results.

### 3.6. Gaze and Pupil Metrics vs. Subjective and Performance Metrics

In addition to the growing use of autonomic and gaze metrics, performance measures and subjective assessments are more traditional methods used to evaluate the task difficulty. Many of the reviewed studies used all three types of assessment [9,11,14,16,18,19,20,21,22,28]. The performance measures included game score, accuracy, time to complete, number of attempts, etc. Many subjective assessments were used, including well-established measures such as the NASA Task Load Index (NASA-TLX) [14,16,19,21,22], the Intrinsic Motivation Inventory [20], the Gaming Experience Questionnaire [20], and the Self-Assessment Manikin [18], as well as more recently developed and less common tests, such as Di Stasi’s mental workload test [9]. In some cases, participants were just asked to rate their effort or mental state, their perceived competence, or to compare the current condition to the previous conditions of a task [13,20].

In the reviewed studies, the most commonly used subjective assessment was the NASA-TLX. It was originally developed to assess the subjective workload of operators controlling human–machine interface systems, but has since been widely adopted in many fields. The assessment asks users to rate six dimensions (mental demand, physical demand, temporal demand, performance, effort, and frustration) on a visual scale from ‘very low’ to ‘very high’ [77]. It is now available as a paper and pencil or a computerized test. The assessment manual details the process of determining which subscale is more or less relevant to a specific task and then uses the numerical ratings for each subscale to calculate an overall workload score [78]. Some authors have discussed the weighting of the different dimensions to obtain a composite score [14,22]. Many other authors have chosen to focus on each subscale separately in addition to, or rather than, the composite scores, reporting which subscale showed the most change from condition to condition. For example, Mirandi et al. [22] demonstrated statistically significant increases in total score between the different difficulty levels and reported that the mental and temporal demands were most relevant to their computer task and showed the most change, while the physical demand scores were quite low.

The subjective questionnaires are intrusive to administer mid task. The NASA-TLX manual discusses this potential problem, indicating that, while it may be possible to provide answers in real time, retrospective responses while watching a video replay of the task are not uncommon. Most commonly in the reviewed studies, participants completed the assessment following each task segment or condition [22]. This makes sense as, when considering the concept of the flow state and its emphasis on complete absorption in the task, any attempts to inquire about the subjective experience would interrupt this absorption. However, the subjective recall of engaging tasks may not allow the subject to accurately capture their experience. To increase ease of completion and decrease intrusiveness, some authors chose to use quicker tests instead of, or in addition to, longer assessments, such as the NASA-TLX. One study by Marinescu utilized the Instantaneous Self-Assessment (ISA) at more frequent intervals than the NASA-TLX, though this was still between tasks rather than within tasks [16]. The ISA is a single rating of workload from 1–5 that was proposed for administration mid-task; it has been found to correlate with other post-task subjective assessments, heart rate variability, and performance [79]. In line with this, Marinescu demonstrated strong correlations between mean ISA scores and pupil diameter for the majority of subjects [16]. However, even the ISA, which is designed to limit its impact on the task, can decrease the performance of participants during response times [79]. While subjective measures are one piece of the puzzle, continued understanding of their triangulation with performance and autonomic measures is crucial to refine workload and effort assessment.

In the studies that utilized all three measures, the comparison of performance, subjective, and gaze metrics yielded inconsistent results. Mirandi et al. demonstrated increases in NASA-TLX scores and decreases in performance scores as the difficulty level increased. Consistently, autonomic measures that demonstrated change followed this linear pattern [22]. In line with this, the Index of Cognitive Activity, a pupillary measure, has been shown to correlate with the NASA-TLX, to measure cognitive workload in older adults [80]. Marinescu reported increased NASA-TLX mental demand scale scores and normalized ISA scores with increased difficulty; the ISA scores correlated negatively with performance and positively with pupil diameter [16]. Strauch’s pong study demonstrated an overall inverted U-shaped response for pupil dilation across difficulty levels. Subjective reports of fun/enjoyment and holding attention/focus/effort correlated positively with this and boredom correlated negatively. However, game score decreased consistently across difficulty levels [20]. In Schirm’s VR phonebooth language comprehension task, pupil dilation response was not linear with difficulty level, and subjective ratings of frustration, which sharply increased for the highest difficulty level as the pupil diameter decreased, reflected this as well. The subjective scores for perceived mental demand increased and for perceived comprehension ability decreased linearly as the difficulty increased [19]. Wanyan’s pilot study also demonstrated an inverted U-shaped pupil response to difficulty level but a linear increase in NASA-TLX score and a linear decrease in performance [21]. Liu’s cognitive versus perceptual load study found that increased cognitive load, but not perceptual load, resulted in decreased performance. Likewise, only increased cognitive, not perceptual, load resulted in increased NASA-TLX scores. However, fixation data were sensitive to both the demand types in different ways, demonstrating the value of gaze data to supplement subjective and behavioral performance data and suggesting, yet again, that the type of demand is critical when considering the assessment of workload [14].

The balance between performance, subjective, and physiological metrics is complex and incompletely understood. The use of only subjective and performance metrics leaves gaps in the analysis of effort and workload. Subjective assessments are vulnerable to biases, difficult to administer in real time without being intrusive and impacting performance, and may interrupt engagement, thus making them difficult to report. Performance and behavioral metrics are useful, but do not directly capture the workload or effort. Gaze and pupil metrics add a more individualized, objective dimension to the assessment of cognitive workload and effort.

## 4. Conclusions

The use of pupil and gaze metrics, specifically pupil dilation, gaze fixation frequency and duration, saccade velocity, and blink frequency, demonstrate value as a noninvasive, objective, and real-time method to evaluate the mental workload and effort of an individual during a variety of tasks. While some measures, primarily pupil dilation, appear to change consistently with task effort, others vary, including fixation, saccade, and blink metrics. The fixation frequency and duration results vary across studies, conceivably due to differences in cognitive versus perceptual load across tasks. In terms of saccade measures, saccade velocity, specifically peak velocity, is the most commonly used measure and appears to be sensitive to both increased task difficulty, associated with engagement, and with mental fatigue, associated with a lack of engagement or prolonged time on the task. The blink rate also appears sensitive to increased and decreased arousal, as well as to internally versus externally directed attention and cognitive effort. Pupil dilation, while not the most specific measure, is the most consistent marker of increased cognitive arousal and effort with increases in the task difficulty and can discern when the task load increases past the optimal level of challenge of the individual. Despite the need for further research to refine our understanding, it is clear that this review demonstrates the potential value of these metrics in a rehabilitation setting to inform the initial design and modification of ongoing therapeutic activity.

Many of these measures appear to be linked to the dopaminergic reward system. Increased dopamine levels are associated with increased saccade velocity (which also occurs with task demand that increases arousal), increased blink frequency (which also occurs with tasks that demand internal attention), and increased pupil arousal (which occurs with task effort and arousal across the board). The intrinsic motivation to participate and succeed in gamified and simulated tasks may serve as the reward that drives increased task effort with increased workload, thus causing increased dopamine responses and resulting in these eye and pupil changes. During gamified virtual rehabilitation, the role of feedback and internal and external motivators are a crucial aspect of providing an engaging rehabilitation experience that maximizes recovery. The use of these gaze and pupil metrics could provide clinicians with essential insight to inform task parameters and feedback mechanisms.

Fixation, saccades, and blink rate all appear to be impacted by the specific nature of the task demands. Fixation duration and frequency are sensitive to perceptual and cognitive load, while saccade velocity is sensitive to arousal and blink rate to internal or externally directed attention. That being said, it is often difficult to classify tasks in this way due to a significant overlap in the type of demand, and very few studies attempt to do so. This warrants studies specifically designed to examine this dichotomy. As a newly developing methodology, researchers demonstrate a wide range of equipment and processing protocols that will continue to be refined as the field develops. The integration of newly popular pupil and gaze metrics with more traditional behavioral performance measures and subjective measures yields inconsistent results; in some instances, they complement each other well and, in other instances, they provide an interesting contrast. The complex relationship between physiological, autonomic, and performance metrics warrants further investigation. In addition, many other physiological measures, including heart rate variability, electrodermal activity, respiratory activity, and muscle activity, are utilized to estimate the cognitive, emotional, and psychological state in research settings. Additional research is warranted to compare and contrast the best use of these different metrics to provide the most comprehensive picture of the engagement of an individual in motor rehabilitation.

In summary, there is evidence supporting the use of gaze and pupil metrics in the real-time assessment of effort and the cognitive state. In stroke rehabilitation, the ability to assess patient engagement during therapeutic tasks could inform optimal task design, contributing to the essential dosage, intensity, and salience of therapy that have been proven essential to maximize recovery. This necessitates future high-quality studies exploring the use of autonomic and gaze metrics in individuals with neurologic conditions during rehabilitation.

## Figures and Tables

**Table 1:** Description of task and type of gaze/pupil metrics in each primary study.

Author	Task	Gaze/Pupil Behavior
Chapmen et al. [8]	Word processing in patients with aphasia	pupil
Di Stasi et al. [9]	Gamified firefighting task (healthy adults)	saccades, fixation, pupil
Di Stasi et al. [10]	Driving simulator (healthy adults)	saccades
Gutjahr et al. [11]	Gamified cycling (healthy adults)	pupil
Kahya et al. [12]	Postural balance task (adults with Parkinson’s disease)	pupil
Köles et al. [13]	Tetris (healthy adults)	pupil
Liu et al. [14]	Video game (healthy adults)	fixation
Mallick et al. [15]	Tetris (healthy adults)	saccade, blink, pupil
Marinescu et al. [16]	Computerized game with joystick (healthy adults)	pupil
Mitre-Hernandez et al. [17]	Gamified fractions math game (healthy kids)	pupil
Patel et al. [18]	Computerized tracking and reaction games (healthy adults)	pupil
Schirm et al. [19]	VR phonebooth language comprehension task (healthy adults)	saccade, fixation, blink, pupil
Strauch et al. [20]	Pong (healthy adults)	pupil
Wanyan et al. [21]	Flight simulator (pilots)	pupil
Marandi et al. [22]	Computerized shape recreation task (healthy adults)	saccades, fixation, blink, pupil

**Table 2:** Equipment used in primary studies.

Author	Task + Population	Gaze/Pupil Behavior
Chapmen et al. [8]	Eyefollower 2.0 at 120 Hz	pupil
Di Stasi et al. [9]	EyeLink II (headband) at 500 Hz	saccades, fixation, pupil
Di Stasi et al. [10]	Eyelink II (head-mounted) at 500 Hz	saccade
Gutjahr et al. [11]	faceLAB 5	pupil
Kahya et al. [12]	Tobii Pro 2 (glasses) at 60 Hz	pupil
Köles et al. [13]	Tobii T-120 at 120 Hz	pupil
Liu et al. [14]	EyeLink 1000 at 500 Hz	fixation
Mallick et al. [15]	Tobii Pro TX300 at 300 Hz	saccade, blink, pupil
Marinescu et al. [16]	RED 250 at 60 Hz	pupil
Mitre-Hernandez et al. [17]	EyeTribe at 60 Hz	pupil
Patel et al. [18]	GazePoint GP3 at 60 Hz	pupil
Schirm et al. [19]	HTX Vive Pro (VR headset) (frequency varied)	saccade, fixation, blink, pupil
Strauch et al. [20]	SMI-RED 120 at 60 Hz	pupil
Wanyan et al. [21]	Smart Eye Pro at 60 Hz	pupil
Marandi et al. [22]	Eye-Trac 7 at 360 Hz	saccade, fixation, blink, pupil
